# Photo- and Acid-Degradable Polyacylhydrazone–Doxorubicin Conjugates

**DOI:** 10.3390/polym13152461

**Published:** 2021-07-27

**Authors:** Maria Psarrou, Martha Georgia Kothri, Maria Vamvakaki

**Affiliations:** 1 Department of Materials Science and Technology, University of Crete, Vasilika Vouton, 700 13 Heraklion, Crete, Greece; psarroum@iesl.forth.gr; 2 School of Medicine, University of Crete, Vasilika Vouton, 700 13 Heraklion, Crete, Greece; mayakothris@gmail.com; 3 Institute of Electronic Structure and Laser, Foundation for Research and Technology-Hellas, Vasilika Vouton, 700 13 Heraklion, Crete, Greece

**Keywords:** polyacylhydrazones, main-chain cleavage, photo-degradable polymers, acid-degradable polymers, prodrugs, doxorubicin

## Abstract

Light-mediated polymer degradation has attracted considerable attention in various applications, including photo-patterning, tissue engineering and photo-triggered drug delivery. In this study, we report the synthesis and characterization of a new, linear, main-chain photo- and acid-degradable copolymer based on acylhydrazone linkages. The polymer was synthesized via a step-growth copolymerization of adipic acid dihydrazide with a bifunctional poly(ethylene glycol) bearing benzaldehyde end-groups, under mild acidic conditions, to afford a hydrophilic PEG-*alt*-adipic acid (PEG-*alt*-AA) alternating copolymer. The synthesized polymer was characterized by size exclusion chromatography, proton nuclear magnetic resonance and attenuated total reflection-Fourier transform infrared spectroscopies. The main-chain photo- and acid-induced degradation of the copolymer in dimethylsulfoxide and water, respectively, was verified by UV-vis spectroscopy at light intensities as low as 0.1 mW cm^−2^ at λ = 254 nm. Next, a model anticancer drug, doxorubicin (DOX), was chemically linked to the polymer chain end(s) via acylhydrazone bond(s), resulting in amphiphilic PEG-*alt*-adipic acid-DOX (PEG-*alt*-AA-DOX) polymer–drug conjugates. The conjugates were self-assembled in water to form spherical nanoparticles, as evidenced by scanning and transmission electron microscopies. The irradiation of the self-assembled PEG-*alt*-AA-DOX conjugates with UV light and the decrease of the solution pH resulted in the disruption of the assemblies due to the photolysis and acidolysis of the acylhydrazone bonds, and the release of the therapeutic cargo.

## 1. Introduction

Stimuli-degradable polymers (SDPs) have gained increasing attention due of their wide range of potential applications in various fields, including nano- and bio-technology as drug carriers and actuators, and in bio-patterning, etc [1,2]. Recently, SDPs have also attracted significant scientific and technological interest in the design of new recyclable and sustainable materials to eliminate the concerns of global environmental pollution from the non-degradable plastics [3,4].

Among the various chemical and physical stimuli (e.g., pH, temperature, enzymes, ultrasound) employed to trigger the degradation of the polymer chains, light-degradable polymers have attracted considerable interest. Light, as an external stimulus, offers unique advantages such as spatiotemporal control, as well as the tunability of the irradiation wavelength and intensity [5]. So far, the vast majority of photodegradable polymers have relied on the light-induced cleavage of specific chemical bonds, such as ester, triazine, ketal/acetal and urethane, which are accompanied by certain photo-absorbing moieties, namely o-nitro benzyl (ONB) groups, coumarin derivatives or other aromatic units. The photolabile bonds are usually located either in the pendant groups of the polymer chain or as a single photocleavable bond as a junction point of two long polymer chains. However, the degradation of such materials results in the production of high molecular weight photo-products, which hinder their use in biomedical applications (drug delivery or tissue engineering) in which the removal of the by-products from the living organisms is crucial. On the other hand, photodegradable polymers bearing multiple photo-cleavable groups along their main-chain, to produce small molecule photo-products upon irradiation, are limited [2,6]. In 2011, our group introduced, for the first time, a new class of photodegradable polymers based on poly(ketals/acetals), exhibiting main-chain scission at incredibly low irradiation doses (10 mJ cm^−2^) compared to other polymeric materials [7,8]. More recently, Kowollik and co-workers reported the synthesis of main-chain photodegradable polyurethanes by introducing ONB moieties along the backbone of a polyurethane chain, and studied the degradation profiles of these materials both in solution and on thin films using a nanosecond pulsed laser at 340 nm [9].

SDPs have been extensively explored as nanocarriers for the controlled and/or programmable delivery of drugs and other active biomolecules, exhibiting very promising results. Polymer micelles, polymersomes, nanogels and polymer–drug conjugates are typical nanostructures employed in drug delivery applications [1,10,11,12,13,14,15,16,17]. Among them, polymer–drug conjugates, also known as prodrugs, offer specific advantages over other drug delivery systems. First, the drug is covalently bound either along or at the end of the polymer chain, thus preventing its toxic side effects. Second, the covalent attachment of the drug onto the polymer prevents the passive diffusion of the drug and its leakage from the carrier during blood circulation, which is a common problem in physically entrapped drugs within nanocarriers, resulting in a lower drug accumulation at the tissue of interest. Last, the incorporation of stimuli-degradable drug–polymer linkages allows site-specific drug release and increases its therapeutic efficacy [18,19,20,21,22,23]. Even though there are a few studies on stimuli-activatable prodrugs, polymer–drug conjugates based on a main-chain photodegradation process have been scarcely reported in the literature [24].

Linear, main-chain polyhydrazones and polyacylhydrazones belong to a polymer family that has barely been studied in the literature because of their low solubility in both polar and non-polar solvent media [25,26]. Hydrazones and acylhydrazones are acid-sensitive bonds, typically formed by the reaction of a carbonyl moiety (aldehyde or ketone) with a hydrazine or hydrazide group, respectively, under mild conditions. The hydrazone bond has been extensively employed in the development of acid-degradable polymer-drug conjugates, polymer micelles, hydrogels and other materials [27,28,29,30,31,32]. It is well-known that the pH varies in the different tissues and cellular compartments. In cancer tissues, the microenvironment is slightly acidic, 0.5–1.0 pH lower compared to the normal tissues, due to the increased glycolysis in cancer cells. Moreover, a larger decrease in the pH is observed in the intracellular organelles, ranging from pH 6.3 in the early endosome to pH 4.7 in the lysosome [33]. Another impressive feature of the (acyl)hydrazone bonds is their dynamic character. The (acyl)hydrazone bond is characterized as a dynamic covalent bond because of its ability to reversibly form and break under mild conditions. The cleavage of the (acyl)hydrazone bond produces the initial molecules without the generation of any toxic by-products. Due to this unique property, several research groups have used these covalent bonds in the development of dynamic materials, including self-healing polymers, adaptable polymer networks, shape-memory materials and others [30,34,35,36].

**Scheme 1:**
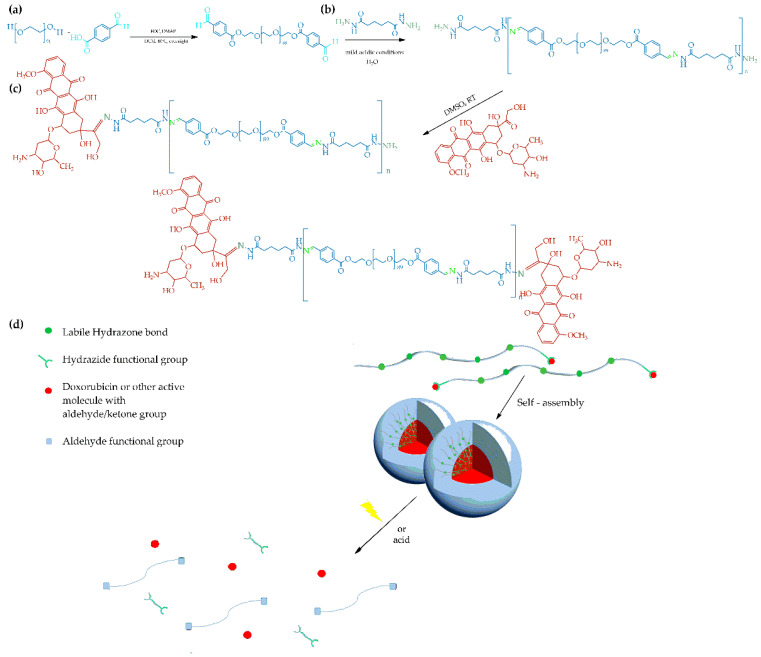
Synthetic routes for the preparation of the (**a**) PEG_ald_ macromonomer, (**b**) PEG-*alt*-AA alternating copolymer and (**c**) PEG-*alt*-AA-DOX drug conjugate. (**d**) Schematic representation of the self-assembly process of the PEG-*alt*-AA-DOX conjugates and the pH- and/or light-induced disruption of the assemblies.

In this study, we designed a linear, main-chain, light-cleavable polyacylhydrazone which was used in the development of photo-degradable polymer-drug conjugates and prodrug nanoparticles. The polymer was synthesized via the step-growth copolymerization of adipic acid dihydrazide with dibenzaldehyde terminated poly(ethylene glycol) (PEG_ald_), under mild acidic conditions, to afford a hydrophilic PEG-*alt*-adipic acid (PEG-*alt*-AA) alternating copolymer (Scheme 1b). The PEG_ald_ macromonomer was prepared by the esterification of HO-PEG-OH with 4-carboxybenzaldehyde (Scheme 1a). The main-chain photo-degradation of the resulting copolymer, upon irradiation at λ = 254 nm with a very low power density, 0.1 mW cm^−2^, in dimethylsulfoxide (DMSO) and water, was investigated. Copolymers with a hydrazide end-group were prepared using an excess of the adipic acid dihydrazide comonomer during the polymerization. Next, the hydrophobic model anticancer drug, doxorubicin (DOX), was linked to the hydrazide end-groups of the polymer via acylhydrazone linkages, to produce amphiphilic PEG-*alt*-adipic acid-DOX (PEG-*alt*-AA-DOX) drug conjugates (Scheme 1c). The conjugates self-assembled in water to form spherical nanoparticles evidenced by scanning and transmission electron microscopies. Following the irradiation of the nanoparticles with UV light, the assemblies were disrupted due to the photolysis of the acylhydrazone bonds along the polymer backbone, and the drug molecules were released. The synergistic action of the two stimuli, pH and light irradiation, augmented the disruption of the self-assembled prodrug nanostructures and the drug release.

## 2. Experimental

### 2.1. Materials and Methods

The 4-carboxybenzaldehyde (98%) and triethylamine (TEA, 99+%) were purchased from Alfa Aesar (Kandel, Germany). The 1-ethyl-3-(3-dimethylaminopropyl) carbodiimide (EDC, ≥97%), 4-(dimethylamino)pyridine (DMAP, 99%), adipic acid dihydrazide (≥98%) and poly(ethylene glycol) (*M*_n_ = 4000 g mol^−1^) were obtained from Sigma Aldrich (Steinheim, Germany) and the doxorubicin hydrochloride salt (DOX∙HCl, >99%) was obtained from LC Laboratories (Woburn, MA, USA). The diethyl ether, petroleum ether, *n*-hexane (96%) and dichloromethane (DCM) were purchased from Scharlau (Sentmenat, Spain). The DCM was dried over calcium hydride. The DMSO (99.9%) was obtained from Fisher Chemical (Loughborough, UK) and the ethanol (≥98%) from Honeywell (Seetze, Germany). Milli-Q water, with a resistivity of 18.2 MΩ·cm at 298 K, was obtained from a Millipore apparatus, and was used for the preparation of all of the samples.

### 2.2. Synthesis of the Dibenzaldehyde Terminated Poly(ethylene glycol) (M_n_ = 4000 g mol^−1^) Macromonomer

First, 4-carboxybenzaldehyde (0.3 g, 2 mmol) and EDC (0.23 g, 1.5 mmol) were dissolved in 20 mL anhydrous DCM, and were stirred at 40 °C for 30 min, followed by the addition of DMAP (0.36 g, 3 mmol) and HO–PEG–OH (2 g, 0.5 mmol). The reaction was stirred at 40 °C for 24 h. Next, the product was precipitated in diethyl ether, washed 10 times with petroleum ether and dried overnight under vacuum at room temperature (RT). The dried product was dissolved in chloroform and filtered through a 0.45 μm PTFE filter to remove the unreacted 4-carboxybenzaldehyde. Finally, it was precipitated again in diethyl ether and dried overnight under vacuum to obtain PEG_ald_ at 82.5% yield.

### 2.3. Synthesis of the PEG-alt-AA Alternating Copolymer

Adipic acid dihydrazide (25 mg, 0.14 mmol) was dissolved in 2 mL milli-Q water. The monomer solution was placed in a sonication bath until its complete dissolution. Next, PEG_ald_ (0.5 g, 0.12 mmol) was added to the solution, followed by the addition of 2–3 drops of 0.1 N HCl. The reaction was left under stirring for 24 h at 25 °C. The next day, the solvent was evaporated using a rotary evaporator, and the reaction was dried under vacuum at RT. The white solid was dissolved in chloroform, and was purified by fractional precipitation in hexane. The final copolymer was stored under a N_2_ atmosphere at 4 °C until use.

### 2.4. Synthesis of a Small-Molecule Acylhydrazone Analogue

Adipic acid dihydrazide (0.1 g, 0.57 mmol) was dissolved in 4 mL ethanol. The solution was first sonicated for 10 min and then stirred for 10 min in a water bath at 40 °C until its complete dissolution. Next, a solution of 4-carboxybenzaldehyde (0.17 g, 1.14 mmol) in 2 mL ethanol was prepared. The two solutions were mixed and a white solid precipitated instantly. The reaction was stirred for another 3 h before filtering off the white solid product and washing it with a large amount of ethanol and water. The final product was dried overnight under reduced pressure at RT.

### 2.5. Synthesis of the PEG-alt-AA-DOX Drug Conjugate

First, DOX∙HCl (2 mg, 0.003445 mmol) was dissolved in DMSO (2.5 mL), and TEA (3 μL) was added to neutralize the HCl. After 20 h stirring at RT, hydrophobic DOX was obtained. Next, the PEG-*alt*-AA copolymer (0.05 g, 0.00179 mmol) was added to the DOX solution, and the reaction mixture was stirred for 20 h before being transferred to a dialysis membrane with a molecular weight cut-off of 3500 Daltons. The mixture was dialyzed against DMSO to remove the unreacted DOX molecules. The conjugated DOX molecules were quantified by fluorescence spectroscopy using a standard calibration curve of DOX in DMSO, and the drug loading was calculated using Equation (1).

(1) $$\%\;\mathrm{Drug}\;\mathrm{loading}=\frac{\mathrm{conjugated}\;\mathrm{DOX}\;\left(\mathrm{gr}\right)\;}{\mathrm{weight}\;\mathrm{of}\;\mathrm{polymer}\;\left(\mathrm{gr}\right)}\times 100$$

### 2.6. Self-Assembly of the Amphiphilic PEG-alt-AA-DOX Drug Conjugates in Water

A solution of the PEG-*alt*-AA-DOX conjugate (5 mg mL^−1^) in 5 mL DMSO was prepared. Next, 15 mL water (pH 7.4) was added to the solution under vigorous stirring at a rate of 0.1 mL min^−1^ using a syringe pump. Afterwards, the solution was dialyzed against water using a dialysis membrane with a molecular weight cut-off of 3500 Daltons to remove the organic solvent. The final solution was filtered through a 0.45 μm pore size cellulose acetate syringe filter, and was kept at 4 °C until use.

### 2.7. Characterization Methods

A Waters size exclusion chromatography (SEC) instrument comprising a Waters 515 isocratic HPLC pump, two Mixed-D and Mixed-E (Polymer labs) columns (Agilent Technologies, Santa Clara, CA, USA, a Waters 2745 Dual absorbance detector and a Waters 410 refractive index (RI) detector (Waters, Milford, MA, USA) were used for the determination of the molecular weight (*M*_n_) and the molecular weight distribution (*M*_w_/*M*_n_) of the polymers. THF (containing 2 *v/v*% triethylamine) was used as the eluent at a flow rate of 1 mL min^−1^. The calibration curve was based on six narrow poly(methyl methacrylate) (PMMA) standards with molecular weights ranging from 625 to 138,000 g mol^−1^. In order to prepare the samples for SEC, the polymers were dissolved in THF at a concentration of 15 mg mL^−1^; the polymer solutions were then filtered through 0.45 μm pore size PTFE syringe filters and injected into the system. ^1^H Nuclear Magnetic Resonance (^1^H NMR) spectra were recorded on two Brucker 300 MHz and 500 MHz spectrometers (Bruker, Rheinstetten, Germany) using CDCl_3_ or DMSO-d_6_ as the solvent. Attenuated total reflection-Fourier transform infrared (ATR-FTIR) spectra were recorded on a Nicolet 6700 spectrometer (Thermo Fisher Scientific, Waltham, MA, USA). For each spectrum, 64 scans were collected in the 500–4000 cm^−1^ range. The ultraviolet–visible (UV-vis) absorption spectra were recorded on a Shimadzu UV-2600 spectrophotometer (Kyoto, Japan) in the wavelength range of 200–600 nm. The samples were prepared by the dissolution of the polymers or polymer–DOX conjugates in DMSO or H_2_O (pH 7.4) at a concentration of 10^−3^ mg mL^−1^. A Malvern Zetasizer Nano ZS instrument (Grovewood Road, UK) equipped with a He–Ne laser (λ = 633nm), at a 90° scattering angle, was used for the dynamic light scattering (DLS) measurements. Before each measurement, the sample was filtered through a 0.45 μm pore size hydrophilic, cellulose acetate syringe filter. The measurements were conducted at 25 °C. The size and the morphology of the self-assembled structures were characterized by field emission-scanning electron microscopy (FE-SEM) using a JEOL JSM-7000F microscope (Tokyo, Japan), and by transmission electron microscopy (TEM) using a JEOL JEM-2100 microscope (Tokyo, Japan). The samples were prepared by the deposition of a drop of a dilute polymer solution on a glass substrate for SEM or a carbon coated copper grid for TEM, and were dried overnight at 25 °C. The fluorescence spectra were recorded on a Lumina Fluorescence Spectrometer (Thermo Scientific) (Waltham, MA, USA) equipped with a 150 W CW Xenon-arc lamp. The excitation and emission slits were set at 10 nm and 20 nm, respectively. The fluorescence spectra of the DOX and the PEG-*alt*-AA-DOX conjugate were recorded from 500 nm to 900 nm upon excitation at 488 nm, whereas the respective spectrum of the copolymer was recorded upon excitation at 302 nm. All of the samples were measured at room temperature.

### 2.8. Photoirradiation Experiments

All of the photoirradiation experiments were performed using a UV lamp at 254 nm and a power density of 0.1 mW cm^−2^. The samples were transferred to a quartz cuvette, and were placed at a distance of 1 cm from the lamp. The photodegradation percentage of the small molecule acylhydrazone, the PEG-*alt*-AA alternating copolymer and the self-assembled PEG-alt-AA-DOX drug conjugate were calculated from the recorded UV-visible spectra by monitoring the decay of the maximum absorption band. For the calculations, the following equation was used:

(2) $$\%\;\mathrm{Photodegradation}=\frac{A_{0}-A_{t}}{A_{0}}\times 100$$

where *A*_0_ and *A_t_* are the maximum absorbance of the acylhydrazone (λ = 302 nm), the PEG-*alt*-AA alternating copolymer (λ = 302 nm) or the PEG-*alt*-AA-DOX conjugate (λ = 297 nm), before and after irradiation for the different time intervals (t), respectively.

### 2.9. Acidic Hydrolysis of the PEG-alt-AA Alternating Copolymer

A 0.2 mg/mL PEG-*alt*-AA alternating copolymer solution in water was prepared; its pH was adjusted at 5.2 using HCl, and the solution was kept under stirring at 37 °C. The hydrolysis of the polymer was monitored by measuring the absorption of the polymer solution at predesigned time intervals by UV-vis spectroscopy.

### 2.10. In Vitro Acid- and Photo-Triggered Drug Release

Aqueous solutions of the self-assembled PEG-*alt*-AA-DOX conjugate (2.5 mg mL^−1^, 3 mL) at pH 7.4, 5.2 and 2.0 were prepared; each solution was transferred to a dialysis bag with a molecular weight cut-off of 3500 Daltons, and was immersed in 10 mL H_2_O at pH 7.4, 5.2 and 2.0, respectively, under gentle stirring (100 rpm) at 37 °C. Next, the samples were irradiated at 254 nm for 5 min at predesigned time intervals. After each irradiation cycle, the solutions were stirred for 15 min, and then the aqueous media were collected and replaced with 10 mL fresh medium of the respective pH value. An aqueous solution of the self-assembled PEG-*alt*-AA-DOX conjugate (2.5 mg mL^−1^, 3 mL) at pH 2.0 was also prepared as a control sample, and the medium was collected and replaced as described above at the predesigned time intervals without prior irradiation. The collected samples were dried, first using the rotary evaporator and then under vacuum for 20 h. The dry samples were dissolved in DMSO (2.5 mL) and their fluorescence spectra were recorded. The concentration of DOX in each sample was determined using a standard calibration curve of DOX in DMSO. The DOX percentage released was calculated using the following equation:

(3) $$\%\;\mathrm{DOX}\;\mathrm{release}=\frac{C_{t}+C_{t-1}}{C_{total}}\times 100$$

where, *C*_t_ and *C_t−_*_1_ are the concentrations of DOX at two successive irradiation time intervals, and *C_total_* is the initial concentration of DOX in the self-assembled conjugate.

## 3. Results and Discussion

### 3.1. Synthesis and Characterization of the PEG-alt-AA Alternating Copolymer

**Figure 1:**
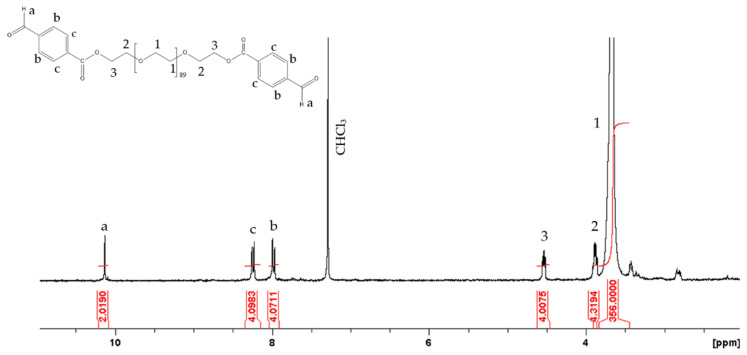
^1^H NMR spectrum of PEG_ald_ in CDCl_3_.

**Figure 2:**
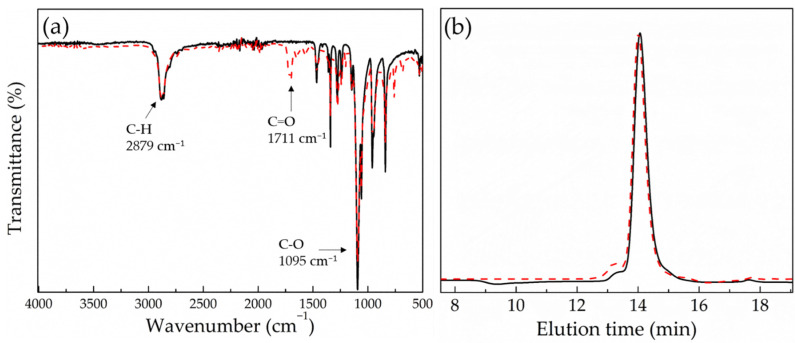
ATR–FTIR spectra (**a**) and SEC traces (**b**) of PEG before (black solid line) and after (red dashed line) esterification with 4-carboxybenzaldehyde.

The main-chain polyacylhydrazones were synthesized via a typical step-growth copolymerization of a dihydrazide and a dialdehyde. Adipic acid dihydrazide was chosen because it is a small, hydrophilic molecule that has previously been used in the biomedical field [37,38]. The second comonomer was a macromonomer, poly(ethylene glycol) (PEG) (*M*_n_ = 4000 gr mol^−1^), which is hydrophilic, non-toxic and FDA approved. Moreover, it is well known for its stealth properties, and has been used extensively in drug delivery [39,40]. In order to introduce the photosensitive moieties and the aldehyde end-groups required to react with the hydrazide functionalities of adipic acid, dihydroxy-functional PEG (HO-PEG-OH) was esterified with 4-carboxybenzaldehyde. The synthesized PEG_ald_ was characterized by ^1^H NMR and ATR-FTIR spectroscopies and SEC. The ^1^H NMR spectrum of PEG_ald_ (Figure 1) showed an intense peak at *δ* 3.67 ppm (H1), which was assigned to the methylene protons of the monomer repeat units of PEG, and two peaks at *δ* 3.87 ppm and *δ* 4.53 ppm (H2 and H3) assigned to the methylene protons next to the newly formed ester bonds, verifying the successful derivatization of the polymer end-groups. In addition, the presence of a peak at *δ* 10.13 ppm, which corresponds to the aldehyde protons (Ha), and the peaks at *δ* 8.0 and 8.24 ppm, attributed to the aromatic protons (Hb and Hc) of the benzaldehyde group, confirmed the presence of the aromatic aldehyde functionalities at the two polymer chain ends. The ATR–FTIR spectrum of the product (Figure 2a) showed the characteristic band of the carbonyl bond (1711 cm^−1^) attributed to the aldehyde and ester groups introduced at the polymer chain ends and supported the NMR results. Finally, the SEC traces of the PEG macromonomer (Figure 2b), before and after esterification with 4-carboxybenzaldehyde, demonstrated the absence of chain degradation during the end-group modification reaction.

**Figure 3:**
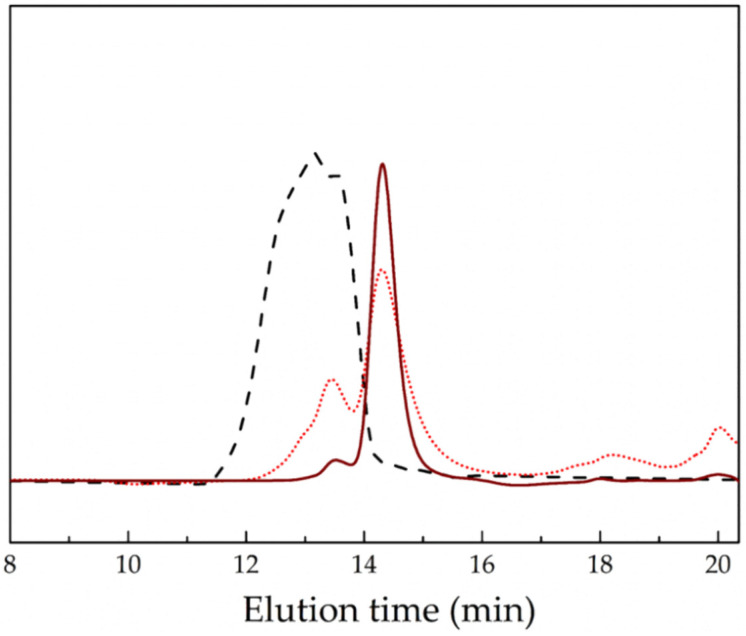
SEC traces of the PEG-*alt*-AA alternating copolymer (black dashed line), the PEG_ald_ precursor macromonomer (wine solid line) and the PEG-*alt*-AA copolymer after 1 h irradiation at 254 nm (red dotted line).

**Figure 4:**
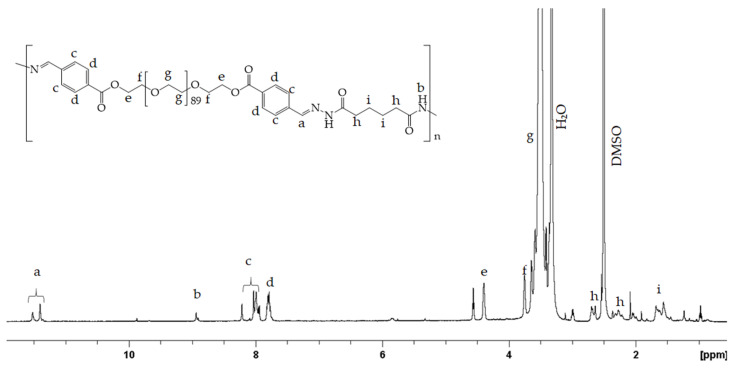
^1^H NMR spectrum of the PEG-*alt*-AA alternating copolymer in DMSO-d6.

**Figure 5:**
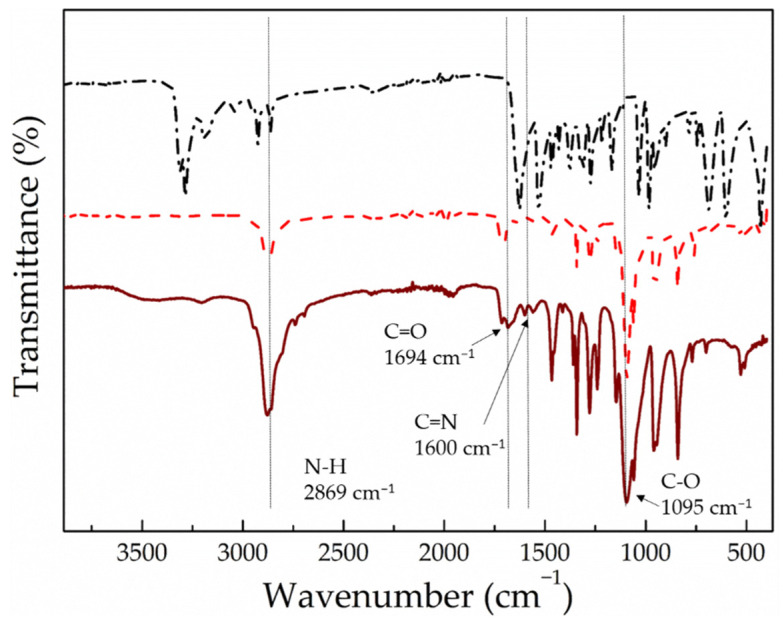
ATR-FTIR spectra of adipic acid dihydrazide (black dashed dotted line), PEG_ald_ (red dashed line) and the PEG-*alt*-AA alternating copolymer (wine solid line).

For the synthesis of the alternating polyacylhydrazone copolymers (PEG-*alt*-AA), the two comonomers—PEG_ald_ and adipic acid dihydrazide—were reacted at a 1:1.2 molar ratio under mildly acidic conditions. The SEC analysis of the polyacylhydrazone polymer prepared (Figure 3) showed a peak which was shifted to lower elution times compared to the PEG macromonomer, verifying the synthesis of a copolymer with *M*_n_ = 28,000 g mol^−1^ and *M*_w_/*M*_n_ = 1.21. The molar mass of the copolymer is quite high and its molecular weight distribution is relatively narrow, given the nature of the condensation polymerization. Moreover, the ^1^H NMR spectrum of the product (Figure 4) showed peaks attributed to the protons of both the PEG and adipic acid comonomer repeat units, as well as the characteristic peak of the hydrazone protons at *δ* 11.42–11.52 ppm (Ha), and confirmed the SEC results discussed above. It should be noted that the absence of the peak of the aldehyde proton of PEG_ald_ at *δ* 10.13 ppm suggests that the quantitative reaction of PEG_ald_ with the dihydrazide comonomer was in excess in the reaction. Figure 5 shows the ATR–FTIR spectra of adipic acid dihydrazide, the PEG_ald_ macromonomer and the PEG-*alt*-AA alternating copolymer. The characteristic vibration bands at 2869 cm^−1^, 1694 cm^−1^ and 1095 cm^−1^—assigned to the N–H, C=O and C–O stretching vibrations, respectively,—were observed in the spectrum of the alternating copolymer. More importantly though, the presence of the characteristic vibration band of the acylhydrazone bond at 1600 cm^−1^ in the spectrum of the copolymer, which is absent in the ATR–FTIR spectra of the two precursor comonomers, verified the successful synthesis of the polymeric product.

### 3.2. Synthesis and Photodegradation of a Small Acylhydrazone Molecule

Before investigating the photodegradation behavior of the PEG-*alt*-AA copolymer, the photosensitivity of the aromatic acylhydrazone bonds was examined using a small diacylhydrazone molecule in a proof-of-concept study. For this, 4-carboxybenzaldehyde was reacted with adipic acid dihydrazide to prepare a diacylhydrazone analogue (Figure S1a). The ^1^H NMR spectrum of the product showed a characteristic peak at *δ* 11.37–11.51 ppm, which was attributed to the acylhydrazone protons, verifying the successful synthesis of the diacylhydrazone derivative (Figure S1b).

**Figure 6:**
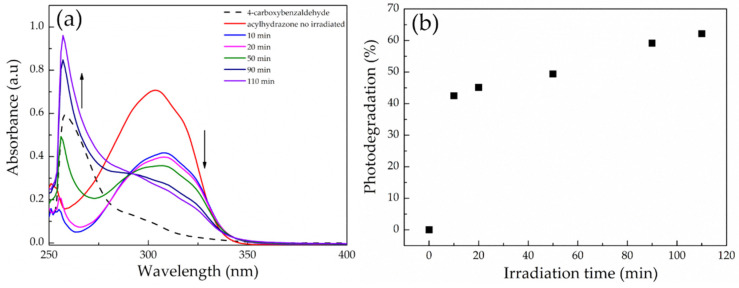
(**a**) UV-vis absorption spectra of a 0.02 mg mL^−1^ solution of the small diacylhydrazone molecule in DMSO upon irradiation at 254 nm, and (**b**) the photodegradation percentage of the diacylhydrazone as a function of the irradiation time.

Next, a solution of the diacylhydrazone molecule in DMSO was subjected to light irradiation (254 nm, 0.1 mW cm^−2^) and its degradation profile was monitored by UV-vis spectroscopy. Before the irradiation, the diacylhydrazone molecule exhibited a broad absorption band centered at ~302 nm (Figure 6a). After the irradiation, the absorbance at 302 nm gradually decreased and a new peak at 254 nm eventually appeared, which coincided with the absorption band of 4-carboxybenzaldehyde, verifying the photo-induced cleavage of the acylhydrazone bonds. The kinetics of the photodegradation process were determined from the decrease in the absorption intensity at 302 nm as a function of the irradiation time (Figure 6b), and showed that ~45% of the acylhydrazone bonds were cleaved after only 10 min of irradiation, whereas the degradation of the bonds was slowed down for longer time intervals, reaching ~65% cleavage after ~2 h irradiation.

### 3.3. Photodegradation of the PEG-alt-AA Alternating Copolymer

Having established the photosensitivity of the aromatic acylhydrazone bonds, the photo-induced degradation of the PEG-*alt*-AA alternating copolymer was investigated using SEC to determine the changes in the polymer molar mass upon light irradiation. As observed in Figure 3, the copolymer peak decreased significantly, and a new peak, which coincides with that of the precursor PEG_ald_ macromonomer, appeared in the SEC trace of the polymer after 1 h irradiation at 254 nm. In addition, a new peak appeared at much higher elution times, indicating the formation of a low-molecular-weight byproduct, which was attributed to adipic acid dihydrazide; however, its size was beyond the analytical range of SEC. These results strongly support the photo-induced cleavage of the acylhydrazone bonds along the main chain of the PEG-*alt*-AA alternating copolymer to produce the two precursor comonomers, PEG_ald_ and adipic acid dihydrazide.

**Figure 7:**
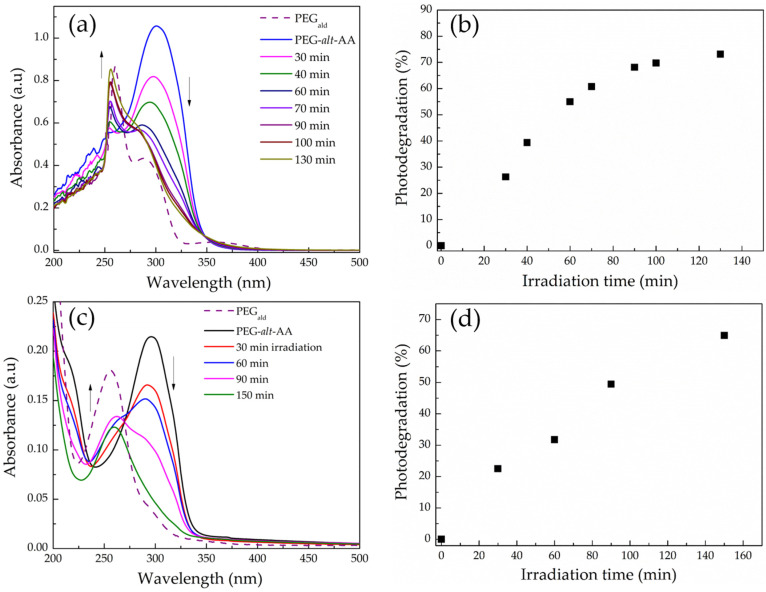
UV-vis absorption spectra of (**a**) a 0.2 mg mL^−1^ copolymer solution in DMSO, and (**b**) a 0.02 mg mL^−1^ copolymer solution in H_2_O at pH 7.4, upon irradiation at 254 nm. The photodegradation percentage of the PEG-*alt*-AA copolymer as a function of irradiation time in (**c**) DMSO and (**d**) H_2_O at pH 7.4.

The light-mediated degradation of the acylhydrazone-based copolymer was further verified by UV-vis spectroscopy. Figure 7a shows the absorption spectra of a 0.2 mg mL^−1^ PEG-*alt-*AA copolymer solution in DMSO. Before irradiation, the polymer peak was centered at ~302 nm, whereas after exposure to UV light, the peak intensity gradually decreased, and two new peaks at 260 nm and 288 nm emerged, which correspond to the absorption bands of the PEG_ald_ macromonomer and support the GPC results discussed above. Figure 7c shows the kinetics of the photodegradation process of the copolymer in DMSO, determined by the UV-vis spectra. An almost-linear degradation profile was found for the first 60 min of irradiation, corresponding to the ~55% cleavage of the polymer bonds, whereas for longer times the degradation kinetics were slowed down and reached 75% cleavage after 130 min irradiation. Similar results were obtained for the photodegradation process of the copolymer in water. The irradiation of an aqueous copolymer solution at pH 7.4, to prevent the acidolysis of the acylhydrazone bonds, led to the decrease of the absorbance at 302 nm, and simultaneously to the appearance of a new peak at 260 nm, which corresponded to the PEG_ald_ macromonomer (Figure 7b). The rate of photodegradation in water was almost linear for more than 2 h of irradiation time (Figure 7d), albeit significantly slower than that found in DMSO, with ~32% photocleavage within the first 1 h of exposure. It is remarkable to mention that despite the fact that the irradiation was performed with UV illumination at 254 nm, which is considered to be a harmful wavelength for biological applications, the irradiation dose used for the complete degradation of the polymer was extremely low (720 mJ cm^−2^) compared to other photodegradable polymers reported in the literature [41,42].

**Figure 8:**
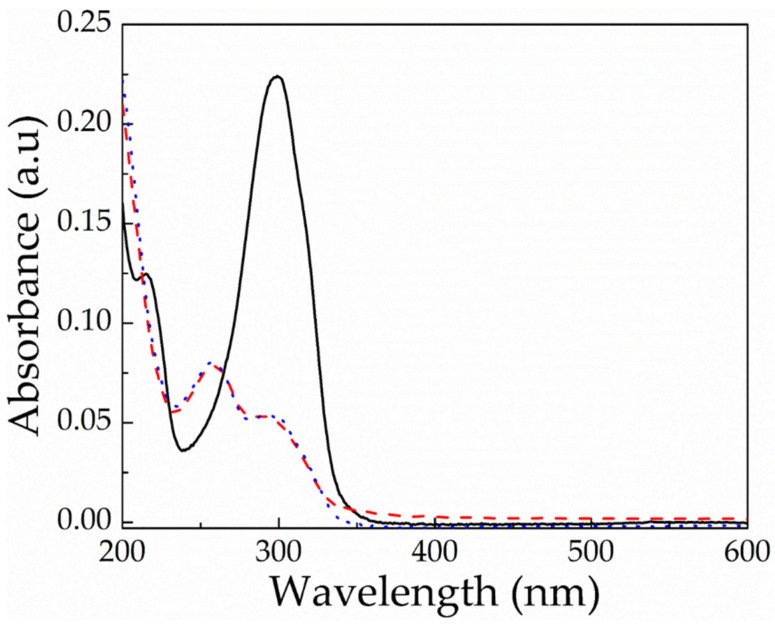
UV-vis absorption spectra of a 0.02 mg mL^−1^ PEG-*alt*-AA alternating copolymer solution in H_2_O before (black solid line) and after (red dashed line) irradiation for 2 h at 254 nm, and subsequently, after stirring for 24 h in the dark (blue dotted line).

**Scheme 2:**
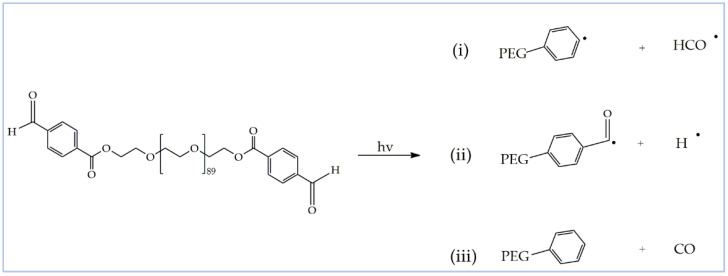
Proposed photodissociation pathways of PEG_ald_ upon irradiation with UV light.

**Figure 9:**
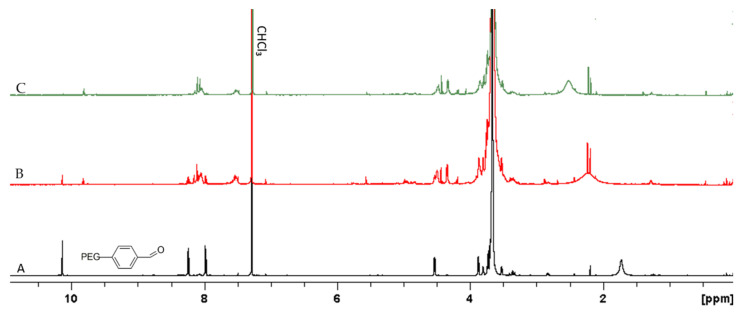
^1^H NMR spectra of PEG_ald_ (**A**) before, (**B**) after 1 h and (**C**) after 2 h irradiation at 254 nm in CDCl_3_.

As discussed above, poly(acyl)hydrazones have been studied in the literature as dynamic covalent polymers, and have been proposed for the development of self-healing materials and adaptable networks due to the reversible nature of the (acyl)hydrazone bonds, which can be cleaved and reformed upon the application of a stimulus, such as acidic media or temperature [34,43,44]. In order to investigate the dynamic nature of the acylhydrazone bonds of the PEG-*alt*-AA copolymer prepared in this work, a solution of the polymer in H_2_O was first irradiated for 2 h at 254 nm to cleave the acylhydrazone bonds, and was afterwards left under stirring for 24 h in the dark. The UV-vis absorption spectra of the copolymer after irradiation and following 24 h in the dark were compared (Figure 8), verifying that no changes in the adsorption took place in the dark, suggesting that the copolymer was not reformed. An explanation for this result could be the sensitivity of the aldehyde groups formed at the PEG chain ends, following the photocleavage of the acylhydrazone bonds by the light stimulus. Aromatic and aliphatic aldehydes (or ketones) are well known to undergo several photochemical reactions upon UV light irradiation [45]. More specifically, the interaction of light with aromatic aldehyde moieties can lead to the formation of different radical species (Scheme 2). In order to further support our hypothesis, ^1^H NMR spectroscopy was used to investigate the fate of the aldehyde end-groups of PEG_ald_ upon UV irradiation. Figure 9 shows that the characteristic peak of the aldehyde protons at *δ* 10.11 ppm decreased significantly after 1 h of irradiation of the PEG_ald_ macromonomer at 254 nm, and disappeared completely after 2 h exposure to UV light. Moreover, the peaks attributed to the aromatic (*δ* 7.99 and *δ* 8.24 ppm) and ester (*δ* 4.53 ppm) protons changed and shifted to lower ppm, whereas two new peaks appeared at *δ* 2.2 ppm and *δ* 9.8 ppm, which were attributed to the formation of acetaldehyde as a by-product of the photodegradation reaction.

### 3.4. Synthesis and Self-Assembly of the PEG-alt-AA-DOX Drug Conjugates

**Figure 10:**
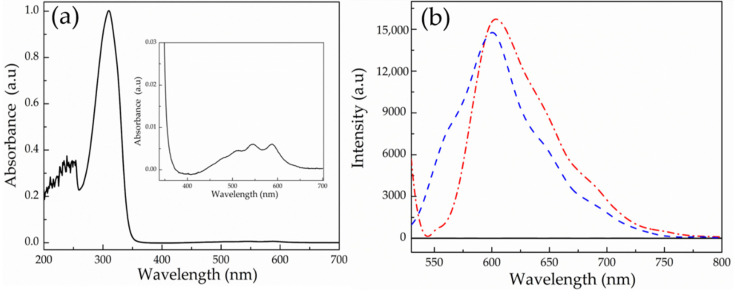
(**a**) UV-vis absorption spectrum of the PEG-*alt*-AA-DOX conjugate in DMSO (inset: magnified spectrum in the 340–700 nm range) and (**b**) fluorescence spectra of the PEG-*alt*-AA copolymer (black solid line), DOX (blue dashed line) and the PEG-*alt*-AA-DOX conjugate (red dashed dotted line).

The PEG-*alt*-AA alternating copolymer was used for the synthesis of the PEG-*alt*-AA-DOX drug conjugates. For the conjugation of DOX at the ends of the polyacylhydrazone chains, the copolymer and DOX were mixed at a 1:2 mole ratio in DMSO and were reacted for 24 h, followed by dialysis against DMSO to eliminate the unreacted DOX molecules. Figure 10a shows the UV-vis absorption spectrum of the PEG-*alt*-AA-DOX conjugate in DMSO, in which the polymer peak at 302 nm is clearly observed, whereas the inset shows the magnified spectrum in the 340–700 nm range, where the absorption peak of DOX is evident, indicating the successful conjugation of the drug at the polymer chain ends. Figure 10b shows the fluorescence emission spectra of the polymer, DOX and the PEG-*alt*-AA-DOX conjugate in DMSO. As expected, the copolymer does not emit light in the 500–800 nm range, whereas DOX and the PEG-*alt*-AA-DOX conjugate both emit at 600 nm, verifying the conjugation of the drug at the polymer chain ends. The drug loading of DOX on the copolymer chains was quantified from the fluorescent intensity of the PEG-*alt*-AA-DOX conjugate using a calibration curve of DOX in DMSO (Figure S2), and was found to be 2.7 × 10^−3^ g DOX/g polymer.

**Figure 11:**
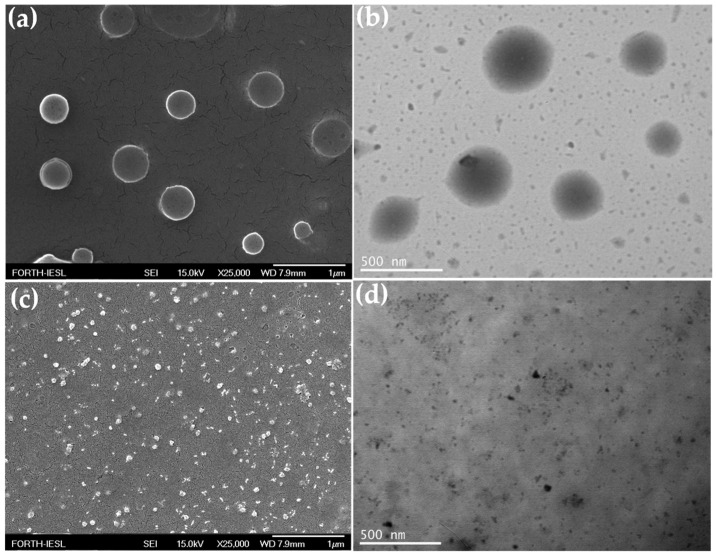
SEM (**a**,**c**) and TEM (**b**,**d**) images of the self-assembled PEG-*alt*-AA-DOX drug conjugates before (**a**,**b**) and after (**c**,**d**) irradiation for 2 h at 254 nm.

**Figure 12:**
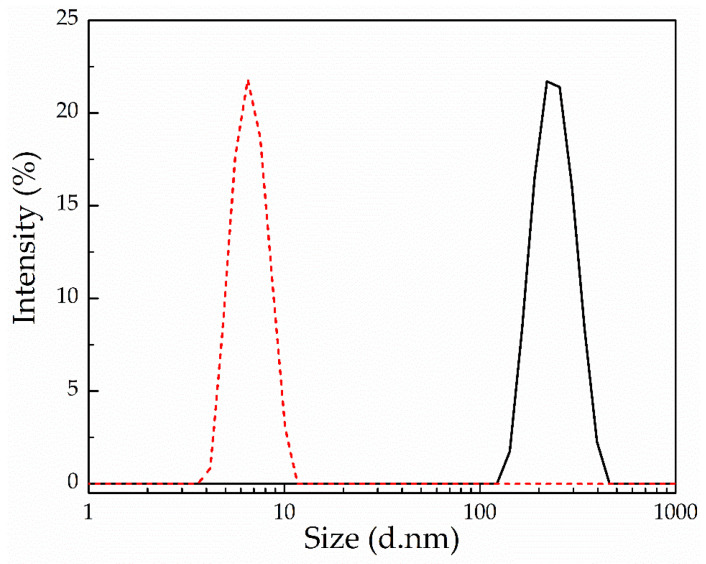
Hydrodynamic diameter for the PEG-*alt*-AA-DOX assemblies in water before (black solid line) and after (red dashed line) irradiation for 2 h at 254 nm.

Amphiphilic molecules containing a hydrophilic and a hydrophobic part can self-assemble into various morphological structures (micelles, vesicles, cylinders, etc.) in water. We envisaged that the conjugation of the hydrophobic DOX drug moieties at one or both ends of the hydrophilic PEG-*alt*-AA copolymer chains, to produce amphiphile drug conjugates, could drive their self-assembly into nanostructures with hydrophilic and hydrophobic compartments. In order to test this hypothesis, the PEG-*alt*-AA-DOX conjugates were self-assembled in water using the dialysis method. First, the polymer-drug conjugates were dissolved in DMSO, which is a good solvent for both the copolymer and the DOX drug, and next, an excess of water was added dropwise to the organic solution, followed by the removal of the DMSO by dialysis. The morphology of the PEG-*alt*-AA-DOX assemblies in water was examined by SEM and TEM microscopies. Interestingly, spherical nanoparticles with an average diameter of ~300 nm were observed by SEM (Figure 11a), and a similar size (~300 nm) was found by TEM (Figure 11b). The hydrodynamic size of the nanoparticles in water was found to be 250 nm by DLS (Figure 12), in good agreement with the SEM and TEM results.

The photo-mediated disruption of the self-assembled PEG-*alt*-AA-DOX nanoparticles and the release of DOX, upon the cleavage of the acylhydrazone bonds with UV light, was investigated by SEM, TEM and DLS. Figure 11c shows an SEM image of the self-assembled PEG-*alt*-AA-DOX conjugates in water (pH 7.4) following irradiation with UV light for 2 h. The absence of the PEG-*alt*-AA-DOX assemblies after irradiation is evident, indicating the main-chain cleavage of the polyacylhydrazone backbone. Smaller nanoparticles, 25–30 nm in size, were observed in the SEM image after light irradiation, which were attributed to aggregates formed by the released hydrophobic DOX molecules. The results were also confirmed by TEM (Figure 11d), which showed the absence of the spherical PEG-*alt*-AA-DOX assemblies, and the presence of small, dark-colored nanoparticles (D~15–20 nm) attributed to the drug aggregates. Finally, the disruption of the self-assembled PEG-*alt*-AA-DOX nanostructures, upon exposure to UV light, was verified by DLS (Figure 12). The large particles disappeared and a hydrodynamic diameter of ~6.5 nm was measured, which was attributed to the constituent PEG macromonomer chains that remained in the solution after the degradation of the polymer backbone.

### 3.5. Photo- and Acid-Induced Disruption of the Self-Assembled PEG-alt-AA-DOX Nanoparticles and the Release of the Drug

**Figure 13:**
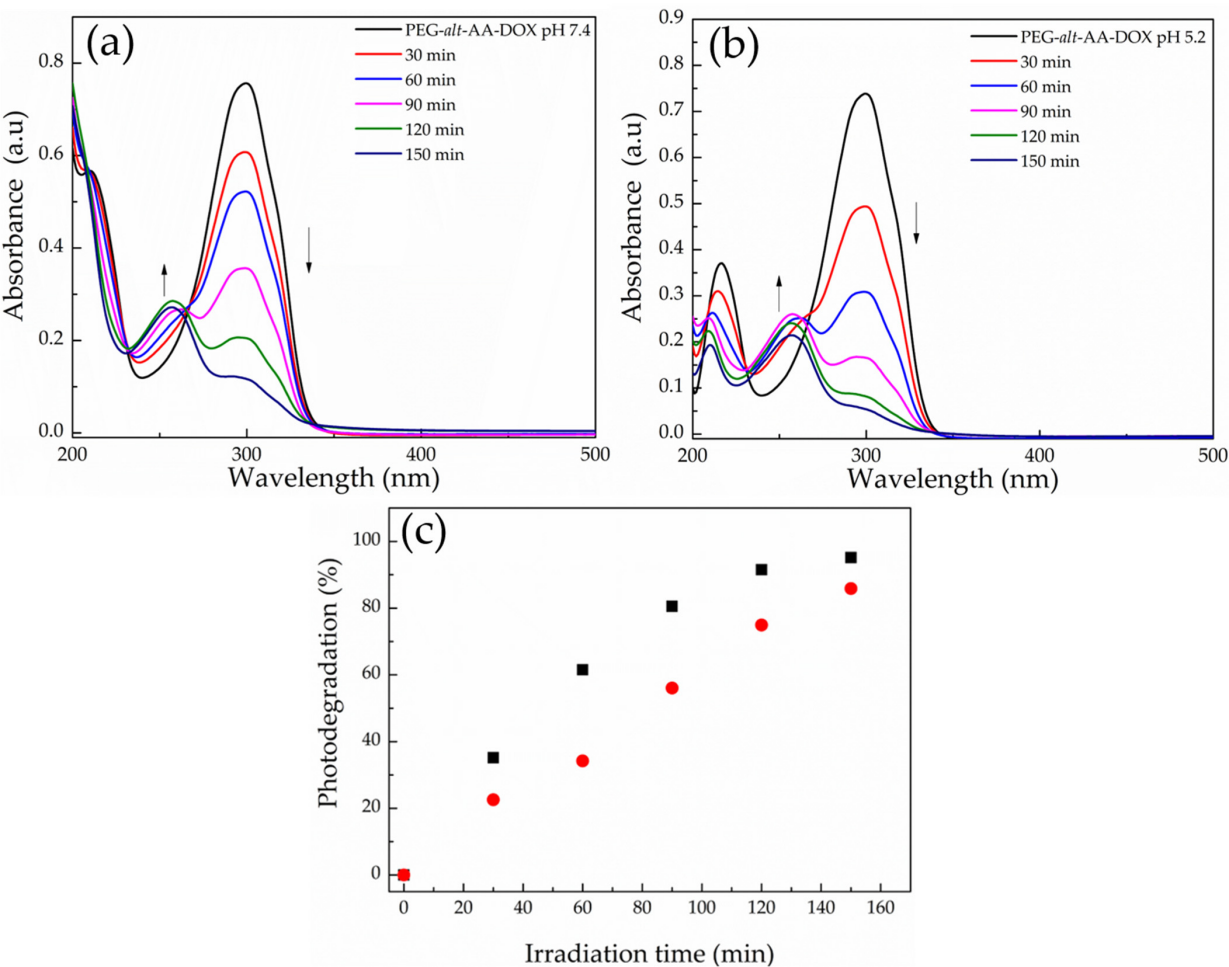
UV-vis absorption spectra of an aqueous PEG-*alt*-AA-DOX solution at (**a**) pH 7.4 and (**b**) pH 5.2 for different irradiation intervals at 254 nm. (**c**) Photodegradation percentage of the PEG-*alt*-AA-DOX nanoparticles as a function of irradiation time at pH 7.4 (red dots) and pH 5.2 (black squares).

**Figure 14:**
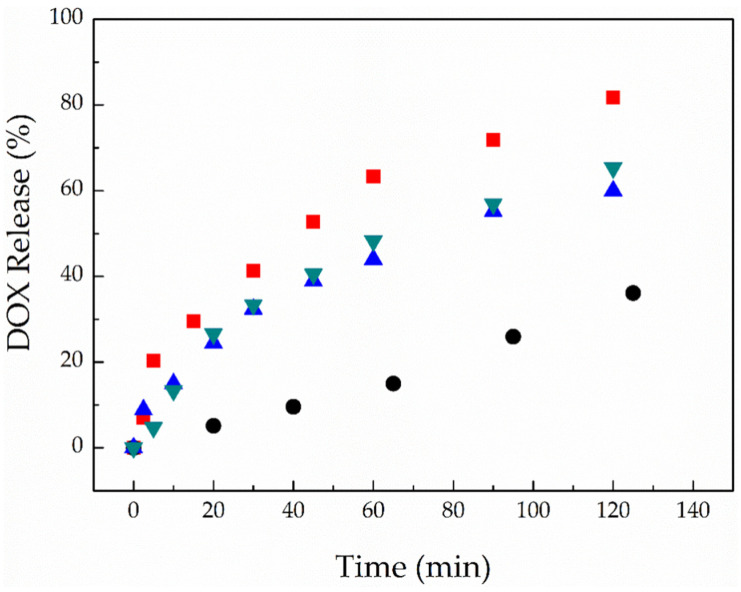
Time-dependant release profile of DOX from the PEG-*alt*-AA-DOX assemblies upon irradiation (254 nm, 0.1 mW cm^−2^) at pH 7.4 (blue triangles), pH 5.2 (green triangles) and pH 2 (red spuares), and the control sample (without irradiation) at pH 2 (black circles).

Finally, the effect of light and a low solution pH on the disruption of the PEG-*alt*-AA-DOX assemblies was studied. Figure 13a shows the UV-vis absorption spectra of an aqueous PEG-*alt*-AA-DOX solution at pH 7.4, for different irradiation times at 254 nm (0.1 mW cm^−2^). As discussed above, the characteristic absorption band of the polymer at ~302 nm decreased with the irradiation time, while a new band at ~260 nm emerged, indicating the main-chain cleavage of the polymer chains and the formation of the precursor PEG macromonomer. A similar degradation process was found for the PEG-*alt*-AA-DOX assemblies upon the irradiation of a polymer solution adjusted to pH 5.2 (Figure 13b), whilst, when comparing the degradation rates at the two pH values, a slightly faster degradation of the polymer—and thus disruption of the assemblies—was found at the low solution pH. In particular, 75% of the polymer was cleaved after 2 h irradiation at pH 7.4, whereas the degree of degradation reached 90% for the same irradiation time at pH 5.2 (Figure 13c), suggesting the synergistic photo- and acidolysis of the labile bonds of the polymer. It is noted that the low DOX content of the PEG-*alt*-AA-DOX assemblies prohibited the quantification of the photo-induced drug release by UV-vis spectroscopy, and fluorescence spectroscopy was used instead. For this, an aqueous PEG-*alt*-AA-DOX solution was first adjusted to pH 5.2 to simulate the acidic environment of a tumor tissue, and was next irradiated with UV light for different irradiation intervals, up to 2 h total irradiation time. The solution was dialyzed against water (pH 5.2) during the irradiation; the dialysate was collected after each irradiation cycle, and its DOX content was analyzed by fluorescence spectroscopy. In parallel, another PEG-*alt*-AA-DOX solution at neutral pH 7.4 was irradiated with UV light, and the release of the cargo in the dialysate was followed by fluorescence spectroscopy. As shown in Figure 14, the release kinetics of the conjugated DOX molecules upon irradiation at pH 5.2 and pH 7.4 were similar (60–65% released DOX at 2 h), denoting that the release of the drug is dominated by the photoinduced cleavage of the acylhydrazone bonds, whereas the solution pH, in this range, does not play a significant role in the drug release profile at short irradiation times (2 h). To support our results, we investigated the degradation rate of the polymer at pH 5.2 as a function of time by UV-vis spectroscopy (Figure S3), and found that the polymer hydrolysis is rather low in the first 2 h (Figure S3 inset) and becomes significant only after 23 h. The slow acidolysis of the hydrazone bonds and the low amount of released cargo at pH 5.0 and 5.5 has also been reported in the literature [22,27,46]. In order to speed up the release kinetics of DOX from the PEG-*alt*-AA-DOX assemblies, an aqueous PEG-*alt*-AA-DOX solution at pH 2.0 was irradiated with UV light for 2 h. Indeed, a faster drug release profile was found (Figure 14, red squares), with 64% of the DOX being released after 1 h irradiation and reaching 82% free drug after 2 h irradiation. On the contrary, the solution pH alone (pH 2 without light irradiation) resulted in only 35% drug release in 2 h (black circles). Based on the above results, light irradiation is a convenient and remote stimulus, allowing to control the drug release from the PEG-*alt*-AA-DOX assemblies in short times, whereas the solution pH enables the prolonged release of the cargo, except for very low pH values (pH 2.0), at which a synergistic photolysis and acidolysis of the prodrug leads to faster release kinetics. Therefore, the proposed drug delivery system could be used for the effective photo-triggered drug release at specific sites/tissues, in which the pH ranges from neutral (normal tissues) to acidic (cancer tissues), providing an additional level of control over the drug release profile. Further studies are underway to investigate the in vitro effectiveness of the proposed polymer–drug conjugates towards cancer cells.

## 4. Conclusions

In summary, a new, main-chain photo- and acid-degradable, amphiphilic polymer–drug conjugate, based on a hydrophilic polyacylhydrazone chain and a hydrophobic model anticancer drug, DOX, was proposed. The alternating polyacylhydrazone copolymer was synthesized via a step-growth polymerization reaction of adipic acid dihydrazide with dibenzaldehyde-terminated PEG. The photodegradation profile of the copolymer—PEG-*alt*-AA—in DMSO and water, upon irradiation at 254 nm (0.1 mW cm^−2^), was studied, and the cleavage of the hydrazone bonds along the polymer main-chain to produce the two precursor comonomers was verified at very low irradiation doses (720 mJ/cm^−1^). Next, amphiphilic PEG-*alt*-AA-DOX conjugates were prepared by the condensation of DOX molecules at the polymer chain end(s), and the conjugates were self-assembled in water to form spherical nanoparticles. At neutral and slightly acidic pH values (pH 5.2), the PEG-*alt*-AA-DOX assemblies were disrupted, and the conjugated drug was released at shorter irradiation times and a low total irradiation dose, whereas the drug-release kinetics were accelerated upon the synergistic effect of light irradiation and a low solution pH (2.0), denoting the remotely controlled drug release nanosystem. We envisage that such photo- and acid-degradable polymers could be used for the conjugation of both hydrophilic and hydrophobic drug molecules, vitamins or other bioactive molecules bearing an aldehyde or ketone group, and are thus attractive for the development of diverse drug carriers. Current studies are underway to investigate the effectiveness of the polymer–drug conjugates towards cancer cells, and to red-shift the irradiation wavelength required for the photodegradation of the polymer acylhydrazone bonds and therefore improve their potential in the biomedical field.

## Data Availability

The data presented in this study are available on request from the corresponding author.

## Supplementary Materials

The following are available online at https://www.mdpi.com/article/10.3390/polym13152461/s1. Figure S1: (a) Schematic representation of the synthetic procedure followed for the preparation of the small diacylhydrazone molecule, and (b) ^1^H NMR spectrum of the product in DMSO-d_6_. Figure S2: (a) Fluorescence emission spectra of DOX as a function of the drug concentration in DMSO, and (b) standard calibration curve of DOX. Figure S3: UV-vis absorption spectra of an aqueous PEG-*alt*-AA solution at pH 5.2 at different time intervals.
