# Supplementary material for: Photo- and Acid-Degradable Polyacylhydrazone–Doxorubicin Conjugates
Source: Polymers (Basel). 2021 Jul 27;13(15):2461. doi: 10.3390/polym13152461 (PMC8348397; doi:10.3390/polym13152461)
Supplement: Supplementary file 1 [file polymers-13-02461-s001.zip › polymers-1231525-supplementary.pdf]

polymers

MDPI

Supplementary Materials

Photo- and Acid-Degradable Polyacylhydrazone-Doxorubicin Conjugates

Maria Psarrou [1], Martha Georgia Kothri [2] and Maria Vamvakaki [1,3,*]

[1] Department of Materials Science and Technology, University of Crete, Vasilika Vouton, 700 13 Heraklion, Crete, Greece
[2] School of Medicine, University of Crete, Vasilika Vouton, 700 13 Heraklion, Crete, Greece
[3] Institute of Electronic Structure and Laser, Foundation of Research and Technology-Hellas, Vasilika Vouton, 700 13 Heraklion, Crete, Greece
* Correspondence: vamvakak@iesl.forth.gr, Tel.: +30-2810-545019

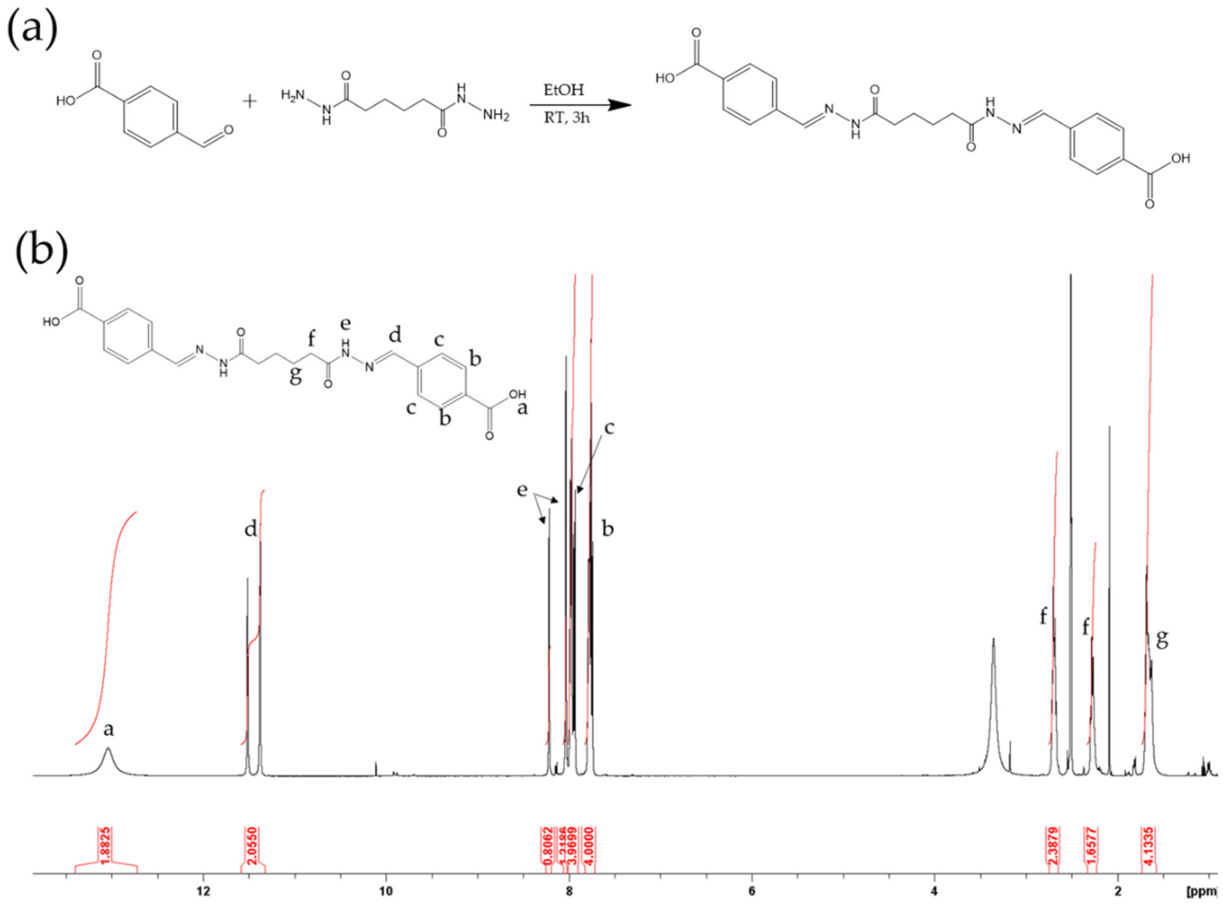

Figure S1. (a) Schematic representation of the synthetic procedure followed for the preparation of the small diacylhydrazone molecule and (b) ^1H NMR spectrum of the product in DMSO-d_6.

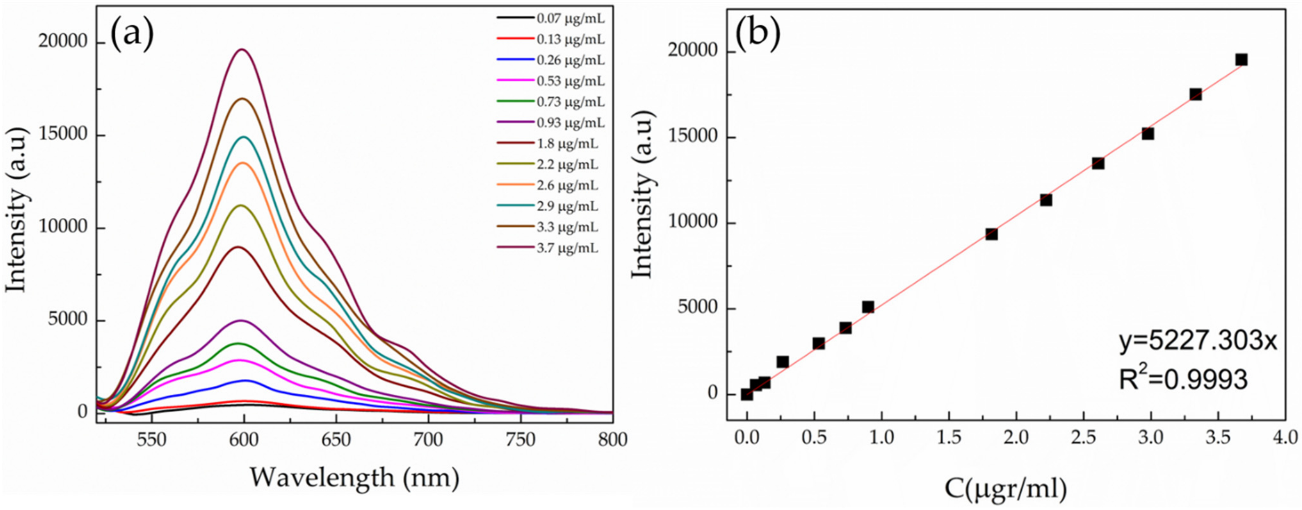

Figure S2. (a) Fluorescence emission spectra of DOX as a function of the drug concentration in DMSO and (b) standard callibration curve of DOX.

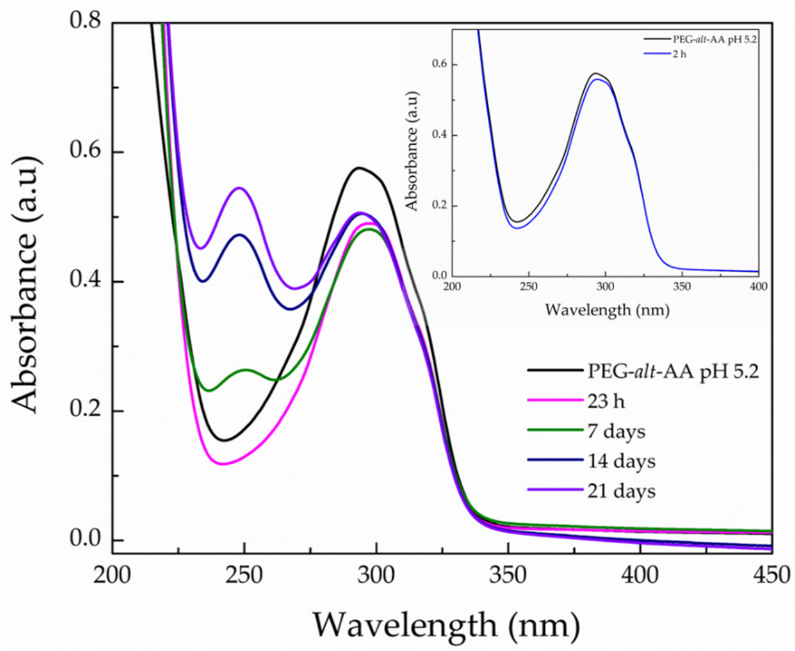

Figure S3. UV-vis absorption spectra of an aqueous PEG-*alt*-AA solution at pH 5.2 at different time intervals. The inset shows the spectra at t = 0 and after 2 h stirring at 37 °C.
